# Red Cell Distribution Width in Relation to Incidence of Stroke and Carotid Atherosclerosis: A Population-Based Cohort Study

**DOI:** 10.1371/journal.pone.0124957

**Published:** 2015-05-07

**Authors:** Martin Söderholm, Yan Borné, Bo Hedblad, Margaretha Persson, Gunnar Engström

**Affiliations:** Cardiovascular Epidemiology Research Group, Department of Clinical Sciences, Lund University, Malmö, Sweden; Tufts University, UNITED STATES

## Abstract

**Background:**

Increased red cell distribution width (RDW) has been related to poor prognosis in patients with cardiovascular disease, and is a predictor of cardiovascular mortality in the general population. The purpose of the present study was to investigate if RDW is associated with increased incidence of stroke and its subtypes in individuals from the general population.

**Methods:**

Red cell distribution width was measured in 26,879 participants (16,561 women and 10,318 men aged 45–73 years) without history of coronary events or stroke, from the population-based Malmö Diet and Cancer Study. Incidences of total stroke and stroke subtypes over a mean follow-up of 15.2 years were calculated in relation to sex-specific quartiles of RDW. The presence of carotid plaque and intima–media thickness, as assessed by ultrasound, was studied in relation to RDW in a randomly selected subcohort (n = 5,309).

**Results:**

Incidences of total stroke (n = 1,869) and cerebral infarction (n = 1,544) were both increased in individuals with high RDW. Hazard ratios (HRs) in the highest compared to the lowest quartile were 1.31 for total stroke (95% confidence interval [CI]: 1.11–1.54, p for trend = 0.004) and 1.32 for cerebral infarction (95% CI: 1.10–1.58, p for trend = 0.004) after adjustment for stroke risk factors and hematological parameters. The adjusted HR for intracerebral hemorrhage (n = 230) was 1.44 (95% CI: 0.90–2.30) and the HR for subarachnoid hemorrhage (n = 75) was 0.94 (95% CI: 0.43–2.07), in the highest compared to the lowest quartile of RDW. Red cell distribution width was positively associated with intima–media thickness of the common carotid artery (p for trend = 0.011).

**Conclusions:**

Red cell distribution width in the highest quartile was associated with increased incidence of total stroke and cerebral infarction. There was no significant association between RDW and incidence of intracerebral or subarachnoid hemorrhage.

## Introduction

The red blood cell distribution width (RDW) describes the variation in red blood cell size (anisocytosis), and is routinely used in the clinical work-up of anemia. Red cell distribution width can be increased in conditions causing a higher proportion of either large or small red blood cells, compared to the individual’s average red cell volume.[1] Increased RDW has been associated with cardiovascular and all-cause mortality in individuals from the general population,[2–4] as well as in patients with heart failure,[5,6] prior myocardial infarction,[7] or stroke.[8,9] In addition, high RDW has been associated with increased incidence of heart failure,[10] atrial fibrillation,[11] and reduced risk of diabetes[12] in middle-aged individuals from the general population.

The reason for the association between RDW and mortality is still unknown, and it is unclear whether RDW is associated with increased incidence of stroke. However, there are several factors that could potentially link RDW to the risk of stroke. For example, activation of the renin–angiotensin system leads to increased erythropoiesis and increased RDW.[13] Activation of the renin–angiotensin system could also increase the risk of ischemic stroke and intracerebral hemorrhage (ICH), for example by increasing the blood pressure.[14,15] Systemic inflammation is related to RDW,[3,4] and is also a risk factor for ischemic stroke.[16] It is not known whether inflammation contributes to hemorrhagic stroke.[16–18] One study suggests that RDW is associated with the presence of carotid plaque and carotid intima–media thickness (IMT).[19] If this association remains even after adjustment for cardiovascular risk factors, it could provide one link between RDW and stroke.[20,21]

The association between RDW and stroke in the general population has not been thoroughly investigated. In a population-based cohort study from Taiwan, Chen et al.[2] found a positive but non-significant association between RDW and stroke. In data from the National Health and Nutrition Examination Survey (NHANES), high RDW was associated with prevalence of stroke, but stroke incidence was not evaluated.[8] Hence, it is still unclear if RDW is associated with incidence of stroke in the general population.

The purpose of the present study was to investigate if RDW is associated with increased incidence of stroke and the subtypes of stroke: cerebral infarction, ICH, and subarachnoid hemorrhage (SAH). In addition, we wanted to assess if atrial fibrillation or carotid atherosclerosis might mediate the association between RDW and ischemic stroke.

## Methods

Between 1991 and 1996, all men aged 46–73 years and all women aged 45–73 years in the city of Malmö, Sweden, were invited to participate in the Malmö Diet and Cancer (MDC) Study by mail and via a newspaper advertisement.[22] A total of 28,449 individuals (11,246 men and 17,203 women) responded, out of an eligible population of 74,138. Participants underwent a physical examination and filled out a self-administered questionnaire. Venous blood samples were collected and a detailed diet assessment was completed as described below.[23]

For the present study, we excluded individuals with prevalent stroke or cardiac events (n = 872) at baseline. We also excluded those who died or emigrated on the day of inclusion (n = 2), due to lack of follow-up. Other exclusions were individuals with missing information about RDW (n = 100), alcohol consumption (n = 389), physical activity (n = 564), smoking (n = 393), body mass index (n = 119), waist circumference (n = 14), or blood pressure (n = 126). After exclusions, the study sample consisted of 26,879 individuals (16,561 women and 10,318 men) with a mean (±SD) age of 58.0±7.6 years.

From the MDC cohort, 6,103 individuals were randomly selected to participate in the MDC cardiovascular cohort (MDC–CC), with the aim of studying the epidemiology of carotid artery disease.[24] Of the present study sample, 5,309 individuals had information on carotid plaque and IMT in the common carotid artery (CCA).

### Ethics statement

All participants gave informed written consent and the study was approved by the ethics committee at Lund University (LU 51/90, 166/2007).

### Baseline examinations

At the physical examination, height, weight, and waist circumference were measured with standard methods. Blood pressure was measured after 10 minutes of rest in the supine position. Information about physical activity, use of tobacco and alcohol, medical history, and current medication was assessed in a self-administered questionnaire. The questionnaire was handed out at the first visit, and was checked for missing values and collected at the second visit, a few weeks later. Eighteen questions concerning different physical activities during different seasons were used to calculate a physical activity score by multiplying the number of minutes per week for each specified activity by an intensity coefficient.[25] Low physical activity was defined as a physical activity score in the lowest quartile. Smoking status was categorized into current (including daily or occasional) smoker, former smoker, and never smoker. Alcohol consumption was categorized into low (<20 g/day for men and <15 g/day for women), intermediate (20–40 and 15–30 g/day), and high (>40 and >30 g/day). Diabetes was defined as self-reported physician-diagnosed diabetes or current use of diabetes medication. History of atrial fibrillation and heart failure at baseline was based on data from the Swedish hospital discharge register. This register was also used to identify those with incident atrial fibrillation during the follow-up period (i.e., individuals with a diagnosis of atrial fibrillation: International Classification of Diseases, 9^th^ revision [ICD9] code 427D, or ICD 10^th^ revision [ICD10] code I48), and to establish the date of diagnosis.[26]

All participants underwent a detailed dietary assessment. An interview-based, modified diet history method was used, combining (1) a 7 day food diary to record intake of meals, beverages, and nutrient supplements; (2) a 168 item questionnaire for assessment of consumption frequencies and portion sizes of regularly eaten foods that were not covered by the food diary; and (3) a 45 minute interview. The methods have been described and validated elsewhere.[23,27] When used as covariates in the present study, iron, folate, and vitamin B12 intakes were log-transformed, standardized for total energy intake, and adjusted for the method of dietary assessment.[28]

### Laboratory tests

Venous blood samples were drawn at the first visit. Hemoglobin, white blood cell count (WBC), and erythrocyte diameter were analyzed in fresh, heparinized blood, using a fully automated assay (SYSMEX K1000 hematology analyzer; TOA Medical Electronics, Kobe, Japan). Red cell distribution width was calculated as the width (fL) of the erythrocyte distribution curve at a relative height of 20% above the baseline. Reference values were 36.4–46.3 fL in women and 35.1–43.9 fL in men.[29,30]

In the MDC–CC, triglycerides and total and high density lipoprotein (HDL) cholesterol were measured from fasting blood samples using standard procedures at the Department of Clinical Chemistry, Malmö University Hospital. The low density lipoprotein (LDL) cholesterol concentration was calculated according to Friedewald’s formula.

### Carotid ultrasound

All participants in the MDC–CC underwent a B-mode ultrasound examination of the right carotid artery for assessment of plaque and measurement of IMT.[24,31] Specially trained and certified sonographers performed the examinations. Plaques were defined as a focal thickening of the intima–media complex >1.2 mm and with an area ≥10 mm^2^, and were scanned for within a predefined area including 3 cm of the distal CCA, the bulb, and 1 cm of the internal and external carotid arteries. Intima–media thickness was measured offline in the far wall of the right distal CCA as the mean thickness over a 10 mm segment proximal to the bifurcation, according to the leading edge principle, using a specially designed computer-assisted analyzing system.[32] The maximum IMT in the carotid bifurcation was also measured. Inter-observer and intra-observer variability of IMT was checked regularly. The mean intra-observer difference was 8.7±6.2% (r = 0.85) and the mean inter-observer difference was 9.0±7.2% (r = 0.77).[31]

### Follow-up and ascertainment of stroke

All participants were followed from the date of the baseline examination until first stroke, death, emigration, or December 31^st^, 2010, whichever came first. Cases with a first stroke were identified from the Malmö stroke registry, which has continuously monitored and searched for stroke cases in both inpatient and outpatient care in Malmö since 1989.[33] Stroke diagnoses were validated by review of the medical records in accordance with the definition from the World Health Organization.[16,34] Diagnosis of cerebral infarction (ICD9 code 434 or ICD10 code I63) was based on computed tomography (CT), magnetic resonance imaging (MRI), or autopsy, either verifying that the infarction location corresponded to the focal neurology or excluding hemorrhage and non-vascular disease. Intracerebral hemorrhage (ICD codes 431 or I61) was considered when CT, MRI, or autopsy showed intraparenchymal blood in the brain. Subarachnoid hemorrhage (ICD codes 430 and I60) was confirmed by CT, lumbar puncture, or autopsy. If neither imaging nor autopsy was performed, the stroke was classified as ‘not otherwise specified’ (NOS) (ICD codes 436 or I64).

Participants in the MDC Study who were treated for stroke in hospitals outside of Malmö were identified from the Swedish hospital discharge register[26] (cerebral infarction: n = 108; ICH: n = 21; SAH: n = 14; NOS: n = 7). This register covers all hospitals in Sweden, and diagnoses are made by physicians in routine care and finally approved by board-certified specialists. Overall, the register covers 99% of hospital discharges and has a high validity.[26] The proportion of correct stroke diagnoses has been shown to be well over 90% in data from 1999[35] and 2003.[36] Nine additional cases with SAH as underlying cause of death were identified in the Swedish cause of death registry; the diagnosis was based on autopsy in six of them. These were considered fatal SAH cases occurring outside of hospital.

### Statistical analysis

Red cell distribution width was categorized into sex-specific quartiles to adjust for the gender differences in RDW and stroke risk. Differences in risk factor distribution across quartiles of RDW were tested using one-way analysis of variance for continuous variables and logistic regression for categorical variables.

Hazard ratios (HRs) and 95% confidence intervals (CIs) for total stroke and stroke subtypes in relation to sex-specific quartiles of RDW were calculated with Cox proportional hazards regression using age as time scale. Models were subsequently adjusted for potential confounders selected *a priori*, based on previous knowledge about risk factors for stroke: systolic and diastolic blood pressure, blood pressure medication, smoking, diabetes, alcohol intake, waist circumference, low physical activity, lipid lowering medication, WBC, and history of atrial fibrillation and heart failure. They were also adjusted for variables strongly related to RDW and previously suggested as confounding factors of the association between RDW and cardiovascular disease:[7] mean corpuscular volume (MCV) and hemoglobin. In addition, we calculated results from a model only including stroke risk factors. Intakes of iron, folate, and vitamin B12 were entered into the model, but since these factors did not have any effect on the association between RDW and stroke, they were left out of the final model.

Effect modification by *a priori* selected variables (sex, age, diabetes, smoking, anemia status, hypertension, and MCV) was evaluated by a likelihood ratio test comparing a model with the interaction term to a model of main effects. The proportional hazards assumption was checked graphically with log–log plots, and by testing for interaction between RDW and time intervals using a likelihood ratio test (three time intervals with approximately equal numbers of events in each interval).

In an additional analysis, we censored subjects diagnosed with incident atrial fibrillation before or concurrent with the stroke event, to investigate the effect of atrial fibrillation on the RDW–stroke association. In this analysis, those with a history of atrial fibrillation at baseline were excluded. This resulted in a model where only subjects without atrial fibrillation contributed follow-up time.

Presence of carotid plaque in relation to sex-specific quartiles of RDW was assessed with logistic regression. Mean IMT was naturally log-transformed due to a right-skewed distribution, and assessed in relation to quartiles of RDW using linear regression. Models were adjusted for (1) age; (2) age and stroke risk factors as listed above, plus HDL and LDL cholesterol and triglycerides (log-transformed due to right-skewed distribution); and (3) model 2 covariates plus MCV and hemoglobin. We found no evidence of multicollinearity for RDW in the full models, as assessed by variance inflation factors.[37]

Statistical analyses were performed with version 12 of the Stata statistical software package (StataCorp, College Station, TX, USA). Incidence rates were calculated using the strate command, which estimates CIs using the quadratic approximation to the Poisson log likelihood for the log rate parameter.

## Results

### Baseline characteristics

Mean RDW (±SD) was 40.5±3.62 fL in men and 40.9±3.30 fL in women. Median RDW was 40.1 fL (interquartile range [IQR] 38.2–42.4) in men and 40.6 fL (IQR 38.6–42.7) in women. As shown in Table 1, participants with a high RDW were more likely to be smokers and high alcohol consumers, and had higher age, MCV, WBC, and vitamin B12 intake. A history of atrial fibrillation and heart failure was slightly more common in those with a high RDW. There were inverse associations between RDW and body mass index, waist circumference, diabetes, and dietary intakes of iron and folate.

**Table 1:** Baseline characteristics by sex-specific quartiles of red cell distribution width (RDW).

	1^st^ quartile	2^nd^ quartile	3^rd^ quartile	4^th^ quartile	P value	All
N	6,689	6,667	6,765	6,758		26,879
Women, %	61.6	61.6	61.9	61.3		61.6
RDW, fL, range, women/men	<38.6/<38.2	38.6–40.5/ 38.2–40.0	40.6–42.7/ 40.1–42.4	>42.7/>42.4		28.2–67.9/ 9.5–123.5
RDW, fL	36.9±1.4	39.4±0.6	41.1±0.7	45.1±2.8		40.7±3.4
Age, yrs	56.8±7.0	57.8±7.4	58.5±7.8	59.1±7.9	<0.001	58.0±7.6
Systolic BP, mmHg	141±20	141±20	141±20	141±20	0.79	141±20
Diastolic BP, mmHg	86±9.7	86±9.9	85±10	85±10	<0.001	86±10
BP medication, %	17.3	16.8	16.3	16.1	0.046	16.6
Hypertension*, %	62.2	59.9	60.9	60.1	0.042	60.8
Waist circumference, cm, women/men	78.9±11/ 94.2±10	78.2±11/ 93.9±10	77.6±10/ 93.5±10	76.4±10/ 92.7±11	<0.001	77.8±11 /93.6±10
Body mass index, kg/m^2^	26.2±3.9	25.9±3.9	25.7±4.0	25.1±4.0	<0.001	25.7±4.0
High alcohol intake†, %	3.2	3.5	4.2	6.4	<0.001	4.3
Diabetes, %	4.5	3.0	2.1	2.0	<0.001	2.9
Lipid medication, %	2.8	2.1	2.5	2.2	0.15	2.4
Low physical activity, %	25.1	24.2	24.0	26.0	0.26	24.8
Smoking						
Current	14.4	21.6	29.6	47.2	<0.001	28.2
Former	37.4	36.1	33.4	26.3	<0.001	33.3
Never	48.2	42.3	37.1	26.5	<0.001	38.5
History of atrial fibrillation	0.6	0.8	1.0	1.1	0.001	0.9
History of heart failure	0.1	0.2	0.1	0.3	0.044	0.2
Hemoglobin, g/L	142±12	142±12	142±12	141±12	0.13	142±12
MCV, fL	86±3.4	88±2.8	90±3.1	93±3.6	<0.001	89±4.2
WBC, 10^9^/L	6.1±1.5	6.3±2.0	6.4±1.8	6.8±3.7	<0.001	6.4±2.4
Iron intake‡, mg/day	15.4	15.3	15.2	15.0	<0.001	15.2
Folate intake‡, μg/day	245	243	239	231	<0.001	239
B12 intake‡, μg/day	5.84	6.03	6.03	6.25	<0.001	6.03
MDC–CC						
n = 4,820	n = 1,325	n = 1,227	n = 1,175	n = 1,093		
HDL cholesterol, mmol/L	1.34±0.4	1.38±0.4	1.41±0.4	1.47±0.4	<0.001	1.40±0.4
LDL cholesterol, mmol/L	4.16±1.0	4.24±1.0	4.19±1.0	4.09 ±1.0	0.005	4.17±1.0
Triglycerides‡, mmol/L	1.25	1.19	1.14	1.12	<0.001	1.18

Values are given as means±standard deviation (SD) or percentages, unless otherwise stated.B12, vitamin B12; BP, blood pressure; HDL, high density lipoprotein; LDL, low density lipoprotein; MCV, mean corpuscular volume; MDC–CC, Malmö Diet and Cancer–cardiovascular cohort; WBC, white cell blood count.

*Blood pressure ≥140/90 or BP medication.

†Men/women >40/>30 g/day.

‡Geometric mean, p value for comparison of log-transformed values.

### Incidence of stroke

During a mean follow-up of 15.2±3.9 years (median 16.1 years, 408,239 person-years), 1,869 incident stroke cases occurred, resulting in a total stroke incidence of 4.58 (95% CI 4.38–4.79) per 1000 person-years. The incidences of cerebral infarction (n = 1,544), ICH (n = 230), and SAH (n = 75) were 3.78 (3.60–3.98), 0.56 (0.50–0.64), and 0.18 (0.15–0.23) per 1000 person-years, respectively.

### Incidence of stroke and stroke subtypes in relation to red cell distribution width

High RDW was associated with increased incidence of total stroke. Participants with an RDW in the highest compared to the lowest quartile had an HR for stroke of 1.31 (1.11–1.54) after full adjustment (Table 2). The effect of RDW on stroke was most apparent for values in the highest quartile of RDW.

**Table 2:** Incidence rates, hazard ratios, and 95% confidence intervals for stroke in relation to quartiles of red cell distribution width.

	1^st^ quartile	2^nd^ quartile	3^rd^ quartile	4^th^ quartile	P for trend
Person-years at risk	105,318	102,757	102,261	97,904	
Total stroke					
N	405	441	447	576	
Incidence rate, /1000 py	3.85 (3.49–4.24)	4.29 (3.91–4.71)	4.37 (3.98–4.80)	5.88 (5.42–6.38)	
HR	1	1.03 (0.90–1.18)	1.00 (0.87–1.14)	1.30 (1.15–1.48)	<0.001
HR*	1	1.03 (0.90–1.18)	0.99 (0.87–1.14)	1.21 (1.06–1.39)	0.008
HR†	1	1.05 (0.92–1.21)	1.04 (0.89–1.20)	1.31 (1.11–1.54)	0.004
Cerebral infarction					
N	332	365	381	466	
Incidence rate, /1000 py	3.15 (2.83–3.51)	3.55 (3.21–3.94)	3.73 (3.37–4.12)	4.76 (4.35–5.21)	
HR	1	1.04 (0.89–1.20)	1.03 (0.89–1.19)	1.27 (1.10–1.46)	0.001
HR*	1	1.04 (0.89–1.20)	1.03 (0.89–1.19)	1.19 (1.02–1.38)	0.028
HR†	1	1.07 (0.92–1.24)	1.09 (0.93–1.28)	1.32 (1.10–1.58)	0.004
Intracerebral hemorrhage					
N	49	56	47	78	
Incidence rate, /1000 py	0.47 (0.35–0.62)	0.54 (0.42–0.71)	0.46 (0.35–0.61)	0.80 (0.64–0.99)	
HR	1	1.10 (0.75–1.61)	0.88 (0.59–1.32)	1.50 (1.04–2.14)	0.055
HR*	1	1.12 (0.76–1.64)	0.90 (0.60–1.35)	1.48 (1.01–2.15)	0.084
HR†	1	1.11 (0.75–1.65)	0.89 (0.57–1.37)	1.44 (0.90–2.30)	0.242
Subarachnoid hemorrhage					
N	18	18	14	25	
Incidence rate, /1000 py	0.17 (0.11–0.27)	0.18 (0.11–0.28)	0.14 (0.08–0.23)	0.26 (0.17–0.38)	
HR	1	1.03 (0.53–1.97)	0.80 (0.40–2.61)	1.48 (0.81–2.73)	0.286
HR*	1	0.93 (0.48–1.80)	0.65 (0.32–1.32)	0.99 (0.52–1.89)	0.824
HR†	1	0.92 (0.47–1.81)	0.63 (0.30–1.35)	0.94 (0.43–2.07)	0.701

HR, hazard ratio; py, person-years.

*Adjusted for systolic and diastolic blood pressure, blood pressure medication, smoking, diabetes, alcohol intake, waist circumference, low physical activity, lipid lowering medication, white blood count, history of atrial fibrillation, and heart failure.

†Adjusted for * plus mean corpuscular volume and hemoglobin.

The incidence of cerebral infarction was also increased in individuals with a high RDW (p for trend over quartiles = 0.001) (Table 2). After full adjustment, the HR for cerebral infarction was 1.32 (1.10–1.58) in the highest compared to the lowest quartile.

The HR for ICH, comparing the highest and the lowest quartile of RDW, was 1.50 (95% CI 1.04–2.14, p for trend = 0.055) after adjustment for age and sex. However, the association was attenuated after adjustment for risk factors for stroke and hematological parameters (Table 2). There was a positive but non-significant association between RDW quartiles and SAH, but the association was attenuated after full adjustments (Table 2).

The association between RDW and incidence of total stroke was modified by smoking status, with somewhat lower HRs in former smokers compared to current and never smokers (Table 3). We found no evidence of effect modification by sex, age, diabetes, anemia status, MCV, or hypertension (Table 3).

**Table 3:** Association between red cell distribution width (RDW) and total stroke in selected subgroups.

	HR* (95% CI) in 4^th^ versus 1^st^ quartile of RDW	P for interaction
Sex		0.60
Women	1.29 (1.05–1.59)	
Men	1.25 (1.01–1.54)	
Age		0.84
<60 yrs	1.35 (1.05–1.72)	
≥60 yrs	1.28 (1.06–1.54)	
Diabetes		0.21
No	1.32 (1.12–1.57)	
Yes	1.18 (0.72–1.93)	
Smoking		0.04
Never	1.47 (1.16–1.86)	
Former	1.17 (0.91–1.49)	
Current	1.49 (1.10–2.01)	
Anemia†		0.42
No	1.29 (1.09–1.52)	
Yes	1.37 (0.63–2.99)	
MCV, fL		0.92
<89.3 (median)	1.38 (1.07–1.78)	
≥89.3	1.22 (0.93–1.62)	
Hypertension‡		0.52
No	1.29 (0.95–1.75)	
Yes	1.31 (1.10–1.57)	

CI, confidence interval; HR, hazard ratio; MCV, mean corpuscular volume.

*Adjusted for systolic and diastolic blood pressure, blood pressure medication, smoking, diabetes, alcohol intake, waist circumference, low physical activity, lipid lowering medication, white cell blood count, atrial fibrillation and heart failure, mean corpuscular volume, hemoglobin.

†Hemoglobin <120 g/L for women and <130 for men.

‡Blood pressure ≥140/90 or current medication.

We also performed an analysis excluding individuals with atrial fibrillation at baseline, and censoring individuals with atrial fibrillation during follow-up at the time of incident atrial fibrillation if this happened before, or concurrently with, diagnosis of stroke. The estimates for the associations between RDW and total stroke (n = 1,576) and cerebral infarction (n = 1,285), respectively, were similar to the results in the main analysis. For cerebral infarction, HRs in the second, third, and fourth compared to the first quartile of RDW were 1.00 (0.85–1.18), 1.04 (0.87–1.23), and 1.23 (1.01–1.50), respectively, after full adjustment (p for trend over quartiles = 0.044).

### Red cell distribution width, carotid plaque, and intima–media thickness

Among individuals with high RDW, carotid plaque was somewhat more prevalent, and IMT in the bifurcation and the CCA was slightly higher (Table 4). After full adjustment, IMT in the CCA was significantly associated with RDW, but IMT in the bifurcation or presence of plaque was not. The association between RDW and CCA–IMT was significant in the highest quartile of RDW (0.025 mm increase on the log scale; 95% CI: 0.007–0.043).

**Table 4:** Association between quartiles of red cell distribution width (RDW) and presence of carotid plaque and carotid intima–media thickness.

	RDW				
	1^st^ quartile	2^nd^ quartile	3^rd^ quartile	4^th^ quartile	P for trend
n	1,465	1,353	1,287	1,204	
**Plaque**					
n (%)	468 (32)	419 (31)	456 (35)	445 (37)	
OR* (95% CI)	1	0.88 (0.75–1.04)	1.06 (0.90–1.25)	1.11 (0.94–1.31)	0.074
OR† (95% CI)	1	0.85 (0.71–1.01)	0.94 (0.79–1.13)	0.89 (0.73–1.08)	0.443
OR‡ (95% CI)	1	0.81 (0.68–0.98)	0.87 (0.72–1.06)	0.78 (0.62–0.99)	0.080
**IMT in the bifurcation**					
Median (interquartile range), mm	1.26 (1.01–1.64)	1.23 (1.02–1.64)	1.28 (1.04–1.73)	1.30 (1.04–1.79)	
Geometric mean, mm	1.31	1.32	1.35	1.38	
Beta* (95% CI)	1	-0.02 (-0.05–0.02)	0.007 (-0.002–0.04)	0.02 (-0.01–0.05)	0.102
Beta† (95% CI)	1	-0.02 (-0.05–0.02)	-0.001 (-0.03–0.03)	-0.001 (-0.04–0.03)	0.852
Beta‡ (95% CI)	1	-0.03 (-0.06–0.08)	-0.02 (-0.05–0.02)	-0.03 (-0.07–0.01)	0.209
**IMT in the common carotid artery **					
Median (interquartile range), mm	0.73 (0.66–0.83)	0.74 (0.66–0.84)	0.75 (0.67–0.84)	0.75 (0.67–0.84)	
Geometric mean, mm	0.74	0.75	0.75	0.76	
Beta* (95% CI)	1	0.004 (-0.009–0.017)	0.004 (-0.010–0.017)	0.008 (-0.006–0.022)	0.277
Beta† (95% CI)	1	0.005 (-0.008–0.019)	0.004 (-0.009–0.018)	0.015 (-0.002–0.029)	0.080
Beta‡ (95% CI)	1	0.009 (-0.005–0.023)	0.011 (-0.004–0.026)	0.025 (0.007–0.043)	0.011

IMT, intima–media thickness; OR, odds ratio; CI, confidence interval; Beta, beta coefficient from linear regression of log-transformed IMT.

*Adjusted for age. N = 5,309 for analyses of plaque and IMT–common carotid artery (CCA), and 3,733 for IMT bifurcation.

†Adjusted for age, systolic and diastolic blood pressure, blood pressure medication, smoking, diabetes, alcohol intake, waist circumference, low physical activity, lipid lowering medication, white cell blood count, history of atrial fibrillation and heart failure, low and high density lipoprotein, triglycerides. N = 4,820 for analyses of plaque and IMT–CCA and 3,393 for IMT bifurcation.

‡ Adjusted for † plus hemoglobin and mean corpuscular volume. N = 4,820 for analyses of plaque and IMT–CCA and 3,393 for IMT bifurcation.

## Discussion

In this population-based cohort study, we observed that incidence of total stroke and cerebral infarction was increased in participants with high baseline RDW, even after adjustment for stroke risk factors and hematological parameters. The results were not explained by prevalent or incident atrial fibrillation, which has previously been associated with high RDW.[11] In addition, we found some evidence for an association between RDW and increased IMT in the CCA, which is a well-known risk factor for ischemic stroke.[20]

Chen et al.[2] evaluated RDW in relation to stroke incidence in the general population, and reported a positive but non-significant association. In our study, the effect of RDW on stroke incidence was moderate, with an approximately 30% increased risk for individuals with RDW in the highest compared to the lowest quartile. The non-significant findings by Chen et al. could be due to low statistical power, since the number of stroke cases (around 200) in that study was considerably lower than in the present study.

Other studies have shown that high RDW predicts mortality in patients with stroke [8,9] and is associated with higher stroke risk in patients with heart failure or coronary heart disease.[6,7] In agreement with results from other studies [10–12], our data show that RDW is also associated with incidence of cardiovascular diseases in the general population. However, this has to be further confirmed.

Because atrial fibrillation is an important risk factor for cerebral infarction, and is also related to RDW levels,[11] atrial fibrillation could hypothetically mediate the association between RDW and cerebral infarction. However, there was still evidence of an association between RDW and stroke when cases with atrial fibrillation were excluded from the analysis. It is therefore unlikely that atrial fibrillation explains the observed association between RDW and cerebral infarction.

It remains unclear why RDW is associated with increased risk of mortality and cardiovascular disease. There are several factors that could hypothetically link RDW with incidence of stroke. Systemic inflammation is associated with future cardiovascular events including cerebral infarction,[16] whereas the association between inflammatory markers and ICH is inconsistent.[16,17] It has been suggested that systemic inflammation constitutes a link between RDW and cardiovascular disease because it disturbs red cell maturation and causes increased RDW.[3,7] In the present study, RDW was associated with WBC, a classic marker of inflammation. However, adjusting for WBC did not substantially reduce the association between RDW and stroke. Even though other measures of inflammation could also increase the risk of stroke, it seems unlikely that the association between RDW and stroke is caused by inflammation alone.

Low plasma levels of folate, vitamin B12, and iron could increase RDW, and nutritional deficiencies could hypothetically explain the association between RDW and stroke. We did not have any information about plasma levels of iron, transferrin, vitamin B12, or folate. However, these biomarkers are not strongly related to stroke, and deficiencies are probably uncommon in this healthy, middle-aged population. Moreover, adjustment for hemoglobin levels, MCV, and intakes of folate, vitamin B12, and iron (i.e., factors associated with deficiency of these nutrients) did not influence the association between RDW and stroke incidence.

Although there was no significant effect modification by MCV, the association between RDW and stroke tended to be stronger in participants with low MCV. This observation suggests that one possible underlying cause of the relationship between RDW and stroke may be a lower erythrocyte turnover and hence a higher erythrocyte lifespan.[12] Erythrocytes become smaller during their lifespan; the MCV of the red cells decreases and RDW increases if the proportion of older cells is larger. Biological functions of the erythrocyte, such as antioxidative mechanisms and cell shape, become impaired over time[38] and the adhesiveness to endothelial cells increases.[39] These changes in erythrocyte function could lead to alterations in hemostasis and thus increase atherosclerotic thrombosis. A recent study which showed a relationship between RDW and risk of venous thromboembolism also suggests a link between prothrombotic effects and high RDW.[40]

Activation of the renin–angiotensin system promotes a prothrombotic state, with effects on the development of atherosclerotic thrombosis.[15,41] It also increases erythropoiesis,[13,42] leading to raised RDW. Increased activation of the renin–angiotensin system could be another possible explanation for the association between RDW and stroke.

Carotid plaque and carotid IMT are strongly related to risk of cerebral infarction.[20,21] One study of hypertensive patients proposed an association between RDW and presence of carotid plaques and higher IMT–CCA, but did not adjust the results for smoking or MCV.[19] Our results suggest that RDW levels are associated with IMT–CCA after extensive risk factor adjustments, but not with presence of plaque or IMT in the bifurcation. Arterial thickening may therefore constitute one link between RDW and stroke. Studies have suggested that IMT in the bifurcation and presence of plaque are predominantly associated with blood lipids, smoking, and ischemic heart disease, whereas IMT–CCA is more related to hypertension.[43,44] We could not find a strong relationship between blood pressure and RDW. However, prevalence of smoking was much higher in subjects with high RDW, which could increase IMT–CCA in this group.

The MDC cohort consists of a representative sample of middle-aged men and women from the general population in Sweden.[45] Since the majority of the population in Malmö is Caucasian, we do not know whether our results are generalizable to non-Caucasians. As also seen in other population-based studies, non-participants in the MDC study had a higher mortality than participants.[45] However, because mortality is strongly and positively associated with RDW, this effect would likely lead to an underestimation of the true relationship between RDW and stroke, if anything.

Several important risk factors for stroke were included in the multivariable Cox model. We used age as the time scale to correct for the higher mean age in subjects with a high RDW, as recommended for cohort studies with arbitrary starting points.[46] Adding age to the covariates did not affect the results. In the analysis of RDW and stroke, we adjusted for prevalence of diabetes (i.e., self-reported physician-diagnosed diabetes or current medication for diabetes) and use of lipid lowering medication. We did not have information about blood glucose, HbA1c, and lipid levels for the entire cohort. Including glucose levels in the definition of diabetes would have given a somewhat higher prevalence. However, diabetes, blood glucose levels, and LDL cholesterol and triglycerides are all positively associated with cerebral infarction, but seem to be inversely associated with RDW (Table 1).[12] Additional adjustment for these factors would likely result in a stronger association between RDW and cerebral infarction.

Although the cohort was large and the number of stroke events high, the numbers of ICH and SAH cases were considerably lower than the number of cerebral infarction events, which is reasonable given their lower incidence in the general population. The statistical power may be too low to detect a significant association for ICH and SAH. These associations should be evaluated in larger samples, and for ICH the evaluation should include consideration of possible differences according to the hemorrhage location.

Red cell distribution width was only measured once at baseline, but may have changed over time due to biological intra-individual variability. However, any change in RDW levels during follow-up would most likely be non-differential and thus bias the results towards the null.

In conclusion, high RDW was associated with increased incidence of total stroke and cerebral infarction in this study from the general population. Red cell distribution width was also associated with carotid IMT, but not with carotid plaque. It remains uncertain how RDW is related to incidence of ICH or SAH. The reasons for the association between RDW and stroke are unclear and should be further investigated.
